# Supplementary material for: Quantitatively assessing early detection strategies for mitigating COVID-19 and future pandemics
Source: Nat Commun. 2023 Dec 20;14:8479. doi: 10.1038/s41467-023-44199-7 (PMC10733317; doi:10.1038/s41467-023-44199-7)
Supplement: Supplementary file 3 — Reporting Summary [file 41467_2023_44199_MOESM3_ESM.pdf]

nature portfolio

Corresponding author(s): Michael Springer, Andrew Bo Liu

Last updated by author(s): Nov 16, 2023

Reporting Summary

Nature Portfolio wishes to improve the reproducibility of the work that we publish. This form provides structure for consistency and transparency in reporting. For further information on Nature Portfolio policies, see our Editorial Policies and the Editorial Policy Checklist.

Statistics

For all statistical analyses, confirm that the following items are present in the figure legend, table legend, main text, or Methods section.

n/a	Confirmed	
[ ]	[x]	The exact sample size (*n*) for each experimental group/condition, given as a discrete number and unit of measurement
[x]	[ ]	A statement on whether measurements were taken from distinct samples or whether the same sample was measured repeatedly
[ ]	[x]	The statistical test(s) used AND whether they are one- or two-sided<br>*Only common tests should be described solely by name; describe more complex techniques in the Methods section.*
[ ]	[x]	A description of all covariates tested
[ ]	[x]	A description of any assumptions or corrections, such as tests of normality and adjustment for multiple comparisons
[ ]	[x]	A full description of the statistical parameters including central tendency (e.g. means) or other basic estimates (e.g. regression coefficient) AND variation (e.g. standard deviation) or associated estimates of uncertainty (e.g. confidence intervals)
[ ]	[x]	For null hypothesis testing, the test statistic (e.g. *F*, *t*, *r*) with confidence intervals, effect sizes, degrees of freedom and *P* value noted<br>*Give P values as exact values whenever suitable.*
[x]	[ ]	For Bayesian analysis, information on the choice of priors and Markov chain Monte Carlo settings
[x]	[ ]	For hierarchical and complex designs, identification of the appropriate level for tests and full reporting of outcomes
[x]	[ ]	Estimates of effect sizes (e.g. Cohen's *d*, Pearson's *r*), indicating how they were calculated

Our web collection on statistics for biologists contains articles on many of the points above.

Software and code

Policy information about availability of computer code

Data collection	Simulation data were generated by code at https://github.com/abliu/early-detection/releases as well as EpiNow2 (v1.3.5).
Data analysis	Analyses and figures were generated by code at https://github.com/abliu/early-detection/releases as well as tidyverse (v1.3.1).

For manuscripts utilizing custom algorithms or software that are central to the research but not yet described in published literature, software must be made available to editors and reviewers. We strongly encourage code deposition in a community repository (e.g. GitHub). See the Nature Portfolio guidelines for submitting code & software for further information.

Data

Policy information about availability of data

All manuscripts must include a data availability statement. This statement should provide the following information, where applicable:

- Accession codes, unique identifiers, or web links for publicly available datasets
- A description of any restrictions on data availability
- For clinical datasets or third party data, please ensure that the statement adheres to our policy

Detection time data are available at https://github.com/abliu/early-detection/releases. Estimated COVID-19 case counts in late 2019 and early 2020 are available from Pekar et al. 2022 (https://github.com/sars-cov-2-origins/multi-introduction) and the US wastewater threshold data are from Wu et al. 2021 (https://www.sciencedirect.com/science/article/pii/S0043135421005984?via%3Dihub#sec0019). In the supplementary analyses, the Massachusetts Water Resources Authority wastewater data are from https://www.mwra.com/biobot/biobotdata.htm, national COVID-19 case counts in early 2020 are from the Johns Hopkins Center for Systems Science and Engineering 2023 (https://github.com/CSSEGISandData/COVID-19), and US state COVID-19 case counts in early 2020 are from Lu et

al. 2021 (https://journals.plos.org/ploscompbiol/article?id=10.1371/journal.pcbi.1008994).

Research involving human participants, their data, or biological material

Policy information about studies with human participants or human data. See also policy information about sex, gender (identity/presentation), and sexual orientation and race, ethnicity and racism.

Reporting on sex and gender	N/A
Reporting on race, ethnicity, or other socially relevant groupings	N/A
Population characteristics	N/A
Recruitment	N/A
Ethics oversight	N/A

Note that full information on the approval of the study protocol must also be provided in the manuscript.

Field-specific reporting

Please select the one below that is the best fit for your research. If you are not sure, read the appropriate sections before making your selection.

☒ Life sciences ☐ Behavioural & social sciences ☐ Ecological, evolutionary & environmental sciences

For a reference copy of the document with all sections, see nature.com/documents/nr-reporting-summary-flat.pdf

Life sciences study design

All studies must disclose on these points even when the disclosure is negative.

Sample size	Sample size was 4,800: 100 simulations for each of 48 system-disease-community combinations. These cover 8 diseases simulated in each of these 6 scenarios: (i) hospital system, (ii-v) wastewater in four different catchment sizes, and (vi) air travel monitoring.
Data exclusions	No data were excluded.
Replication	We validated the model in two independent datasets: 1. we predicted COVID-19 detection times in each US state with a mean absolute error of 0.97 weeks and 2. we found our wastewater-specific results were consistent with the upper bound implied by Massachusetts COVID-19 wastewater data.
Randomization	Simulated detection times were generated with random draws from the following distributions: (i) for each case, whether it was detected or not; (ii) the offspring distribution for each case; and (iii) in the case of wastewater, the distribution of sensitivity thresholds from Wu et al. 2021.
Blinding	Blinding was not applicable because this study is not an experimental trial.

Reporting for specific materials, systems and methods

We require information from authors about some types of materials, experimental systems and methods used in many studies. Here, indicate whether each material, system or method listed is relevant to your study. If you are not sure if a list item applies to your research, read the appropriate section before selecting a response.

Materials & experimental systems

n/a	Involved in the study	
☒	☐	Antibodies
☒	☐	Eukaryotic cell lines
☒	☐	Palaeontology and archaeology
☒	☐	Animals and other organisms
☒	☐	Clinical data
☒	☐	Dual use research of concern
☒	☐	Plants

Methods

n/a	Involved in the study	
☒	☐	ChIP-seq
☒	☐	Flow cytometry
☒	☐	MRI-based neuroimaging
